# High-Frequency Direct Detection of Triazole Resistance in *Aspergillus fumigatus* from Patients with Chronic Pulmonary Fungal Diseases in India

**DOI:** 10.3390/jof6020067

**Published:** 2020-05-20

**Authors:** Ashutosh Singh, Brijesh Sharma, Kaushal Kumar Mahto, Jacques F. Meis, Anuradha Chowdhary

**Affiliations:** 1 Department of Medical Mycology, Vallabhbhai Patel Chest Institute, University of Delhi, Delhi 110 007, India; singh90.ashutosh@gmail.com (A.S.); kausalk@gmail.com (K.K.M.); 2 Department of Medicine, Post Graduate Institute of Medical Education and Research, Dr. Ram Manohar Lohia Hospital, Delhi 110 001, India; brijsushma@gmail.com; 3 Department of Medical Microbiology and Infectious Diseases, Canisius-Wilhelmina Hospital, 6532 SZ Nijmegen, The Netherlands; jacques.meis@gmail.com; 4 Centre of Expertise in Mycology Radboudumc/CWZ, 6532 SZ Nijmegen, The Netherlands

**Keywords:** Azole resistance, *Aspergillus fumigatus*, AsperGenius^®^ multiplex real-time PCR assay, G54, M220, TR_46_/Y121F/T289A, TR_34_/L98H, chronic pulmonary aspergillosis, allergic bronchopulmonary aspergillosis

## Abstract

Aspergillosis due to azole-resistant *Aspergillus fumigatus* is a worldwide problem with major therapeutic implications. In patients with invasive aspergillosis, a low yield of fungal cultures results in underestimation of azole resistance. To detect azole resistance in *A. fumigatus*, we applied the AsperGenius^®^ Resistance multiplex real-time polymerase chain reaction (PCR) assay to detect TR_34_/L98H, and TR_46_/T289A/Y121F mutations and the AsperGenius^®^ G54/M220 RUO PCR assay to detect G54/M220 mutations directly in bronchoalveolar lavage (BAL) samples of 160 patients with chronic respiratory diseases in Delhi, India. Only 23% of samples were culture-positive compared to 83% positivity by *A. fumigatus* species PCR highlighting concerns about the low yield of cultures. Notably, 25% of BAL samples (33/160 patients) had azole resistance-associated mutation by direct detection using PCR assay. Detection of resistance-associated mutations was found mainly in 59% and 43% patients with chronic pulmonary aspergillosis (CPA) and allergic bronchopulmonary aspergillosis (ABPA), respectively. Overall, a G54 mutation, conferring itraconazole resistance, was the predominant finding in 87.5% and 67% of patients with CPA and ABPA, respectively. In culture-negative, PCR-positive samples, we detected azole-resistant mutations in 34% of BAL samples. Azole resistance in chronic *Aspergillus* diseases remains undiagnosed, warranting standardization of respiratory culture and inclusion of rapid techniques to detect resistance markers directly in respiratory samples.

## 1. Introduction

A wide spectrum of pulmonary fungal diseases, including invasive aspergillosis (IA), chronic pulmonary aspergillosis (CPA), *Aspergillus* bronchitis, and allergic bronchopulmonary aspergillosis (ABPA) are primarily caused by *Aspergillus fumigatus* [1]. Azoles are the first line of treatment, independently of the clinical manifestation of disease [2]. The last decade has witnessed several case series of the emergence of azole resistance in clinical settings resulting in increasing rates of treatment failure. [3,4,5]. Azole resistance is often caused by mutations in the hot spot *Cyp51A* gene, implicated in the ergosterol biosynthesis pathway. Largely, two most common groups of resistance in clinical settings have been reported; one based on the environmental route of development of resistance mutations and the second group that develop resistance mutations due to long-term azole therapy [6]. TR_34_/L98H and TR_46_/Y121F/T289A are globally the most prevalent environmental resistance mutations in clinical settings. The second group of resistance mutations consists of point mutations in the *Cyp51A* gene at various positions including G54, M220, P216, G138, and G448 with mutations at locus G54 and M220 being the most common amino acid substitutions in patients on long-term azole therapy [5,7]. Although G54 mutations have predominately been considered to develop de novo, however, *A. fumigatus* environmental isolates harboring G54 mutations have been reported from soil samples of several countries including India, Romania, Tanzania, Germany and Switzerland [8,9,10]. Resistance mutations may be detected directly in respiratory samples via molecular methods thus overcoming the limitations of low sensitivity and long turn-around time that affect culture-based methods. Furthermore, direct detection of azole-resistant mutations in clinical samples may potentially improve clinical management and survival of patients infected with triazole-resistant *A. fumigatus* strains. Several polymerase chain reaction (PCR) assays have been developed that can detect triazole resistance mechanisms [11], and three are commercially available, AsperGenius^®^ (PathoNostics, Maastricht, the Netherlands) [4,12,13,14,15,16,17,18], MycoGenie^®^ (Adamtech, Pessac, France) [19,20,21] and Fungiplex^®^ Aspergillus Azole-R in vitro diagnostics (IVD) PCR (Bruker Daltonik GmbH, Bremen, Germany) [22]. The PathoNostics AsperGenius^®^ Resistance multiplex real-time PCR assay has the ability to detect a range of *Aspergillus* species as well as four environmental resistance-associated mutations (TR_34_ and L98H to detect TR_34_/L98H, T289A and Y121F to detect TR_46_/Y121F/T289A) in the *Cyp51A* gene. Its performance has been validated on bronchoalveolar lavage (BAL) fluid, serum and plasma specimens obtained from cases of suspected IA in haematological and intensive care units (ICU) patients [4,12,13,14,15,16,17,18]. The first report of this commercially available resistance PCR assay, (AsperGenius^®^ PathoNostics, Maastricht, Netherlands) was published in 2015 [12]. The diagnostic performance of the assay was validated in BAL samples including 37 BAL fluid samples from haematology patients and 40 BAL fluid samples from ICU patients [12]. Furthermore, in a multicentre study involving haematological patients with suspected IA the assay has been validated in BAL specimens in a large number of patients (*n* = 201). Interestingly, the study showed that the detection of resistance markers was associated with azole treatment failure [4]. More importantly, in both the above-mentioned studies, this assay detected and differentiated wild-type from resistant strains, even if BAL fluid cultures remained negative [4,12]. Additionally, the detection of mixed infections with azole-resistant and susceptible *Aspergillus* strains was reported in a recent study using AsperGenius^®^ assay on 91 BAL samples obtained from patients with a suspected invasive *Aspergillus* infection [16]. In fact, during culture, mixed resistant and susceptible *A. fumigatus* isolates have been sporadically isolated from the same sample [23,24]. Another study of retrospective detection of azole-resistant *A. fumigatus* in BAL fluid using AsperGenius^®^ assay in 100 patients, with proven or probable IA included 50% patients with cystic fibrosis (CF) and lung transplant patients whereas hematological patients represented only 8% of the cohort [13].

To date, the AsperGenius^®^ assay has been studied in specific patients with underlying hematologic malignancies, CF, lung transplant or other immunosuppressed patients. The high prevalence of resistance markers detected among patients with chronic *Aspergillus* infections in the absence of confirmed cultures has been reported previously using direct PCR for detection of hotspot alteration from respiratory samples of patients in Manchester, UK [25]. Resistance markers were found in 6 of 8 (75%) with ABPA or severe asthma with fungal sensitisation (SAFS), and 10 of 20 (50%) with CPA. This astonishingly high rate of resistance in culture-negative cases has important implications for clinical care. To gain insight in the emerging problem of azole resistance in allergic and chronic aspergillosis patients, we used a commercially available AsperGenius^®^ Resistance assay to directly detect mutations in the *Cyp51A* gene of *A. fumigatus* present in BAL fluid samples obtained predominantly from chronic respiratory diseases patients attending a specialized referral Chest Institute in Delhi, India. AsperGenius^®^ multiplex real-time PCR assay detects the TR_34_, L98H, T289A and Y121F resistance-associated mutations that represent the most prevalent environmental mutation combinations TR_34_/L98H and TR_46_/T289A/Y121F in the *Cyp51A* gene. Considering that G54 and M220 mutations in the *Cyp51A* gene are commonly reported resistance markers in allergic and chronic aspergillosis patients that are frequently on long-term azoles [26], we applied a new PathoNostics AsperGenius^®^ G54/M220 RUO real-time assay that additionally detects G54 and M220 *Cyp51A* mutations directly in BAL specimens.

## 2. Material and Methods

Ethical statement: This study was approved by the Vallabhbhai Patel Chest Institute Ethics committee (Nr. 04.09.2014).

Clinical samples. A total of 160 BAL fluid samples collected consecutively during 2017–2018 from individual patients with chronic respiratory diseases were processed in the Medical Mycology laboratory, Vallabhbhai Patel Chest Institute (VPCI), Delhi, India, to detect azole resistance. No specific inclusion criteria were followed.

1. Mycological cultures. BAL samples were processed for direct microscopic examination by 10% potassium hydroxide (KOH)–Blankophor staining and cultured on two sets each of Sabouraud’s dextrose agar (SDA), one set containing gentamicin and chloramphenicol and the other containing cycloheximide (0.05%), and were incubated at 28 °C and 37 °C for 3 weeks. *Aspergillus* isolates were identified by microscopic and macroscopic morphology and *A. fumigatus* was confirmed by *β tubulin* gene sequencing as described previously [27].
2. Screening for azole resistance and Clinical and Laboratory Standard Institute (CLSI) microbroth antifungal susceptibility testing (AFST): *A. fumigatus* from culture-positive BAL samples were screened for azole resistance. To preliminary screen for azole resistance, a single colony of the *A. fumigatus* was subcultured on Itraconazole (ITC, 4 μg/mL) and Voriconazole (VRC, 1 μg/mL) supplemented SDA plates. All isolates able to grow on at least one of the azole-containing SDA plates were further investigated for their minimum inhibitory concentrations (MICs) by using broth microdilution method according to CLSI M38-Ed3 [28]. The drugs tested included ITC (Lee Pharma, Hyderabad, India, and Janssen Research Foundation, Beerse, Belgium), VRC (Pfizer Central Research, Sandwich, Kent, UK), isavuconazole (ISA, Basilea Pharmaceutica International AG, Basel, Switzerland), and posaconazole (POS, Merck, Whitehouse Station, NJ, USA). Drug-free and mold-free controls were included; microtiter plates were incubated at 35 °C, and MICs were read after 48 h. CLSI-recommended quality control strains, *Candida krusei* ATCC 6258 and *Candida parapsilosis* ATCC 22019, and reference strains, *Aspergillus fumigatus* ATCC 204,305 and *Aspergillus flavus* ATCC 204304, were included. MIC endpoints were defined as the lowest concentration that produced complete inhibition of growth vis-à-vis the hyphal growth in the control well. The AFST results were analyzed by using epidemiological cutoff values (ECVs) of *A. fumigatus* recommended by CLSI M59-Ed2 and are as follows: ITC, VRC and ISA, 1 μg/mL [29].
3. *Cyp51A* gene sequencing and mutation analysis of azole-resistant *A. fumigatus* isolates. DNA extraction of all azole-resistant *A. fumigatus* isolates was done by the phenol-chloroform extraction method. Briefly, *A. fumigatus* spores were scraped and inoculated in 1.5-mL screw-cap tubes with a few glass beads to which 600 µL of extraction buffer (0.2 M Tris-HCl [pH 7.6], 10 mM Ethylenediaminetetraacetic acid (EDTA), 0.5 M NaCl, 1% SDS) was added, followed by bead beating twice for 5 min with a 2-min interval in ice in between. Furthermore, the samples were treated with 700 µL of Tris-saturated phenol-chloroform-isoamyl alcohol (25:24:1), followed by chloroform-isoamyl alcohol (24:1) extraction and ethanol precipitation. The DNA pellet was dried, resuspended in 75 µl of sterile nuclease-free water, and treated with 6 µL (10 mg/mL) of RNase (Sigma-Aldrich, St Louis, MO, USA) for 1 h at 37 °C. DNA was electrophoresed by 0.8% agarose gel electrophoresis including ethidium bromide and was visualized under ultraviolet (UV) light, photographed, and scored manually. PCR amplification of the *Cyp51A* gene was carried out in a 50 µL reaction volume containing 100 ng of genomic DNA, 10 mM (each) forward and reverse primers [8], 2.5 U of Taq DNA polymerase (Invitrogen, Carlsbad, CA, USA), 5 µL of 10× PCR buffer (Invitrogen), and 200 mM deoxynucleotide triphosphate (dNTP) mix (New England BioLabs [NEB], Ipswich, MA, USA). Briefly, the amplified product was purified, followed by sequencing on an ABI 3130XL genetic analyzer (Applied Biosystems, Foster City, CA, USA) using the BigDye Terminator kit (version 3.1, RR-100; Applied Biosystems, Foster City, CA, USA). DNA sequences were analyzed with Sequencing Analysis software version 5.3.1 (Applied Biosystems), and consensus sequences were made using BioEdit software version 7.0.5.3. *Cyp51A* gene sequences were compared with the wild-type susceptible reference *A. fumigatus* strain (Af293).
4. Direct detection of *Aspergillus* species and *Cyp51A* mutations in BAL samples by AsperGenius^®^ Resistance multiplex real-time PCR assay. BAL samples, consecutively collected from patients undergoing bronchoscopy for monitoring or diagnosis in the period 2017–2018 were analysed. Bronchoscopy with BAL was performed at the discretion of the treating physician. Chronic pulmonary aspergillosis patients undergoing bronchoscopy were those with suspicion of aspergillosis, development of pulmonary infiltrates and non-resolving pneumonia, co-existing tuberculosis (TB) or malignancy. BAL fluid samples were collected in complicated ABPA and SAFS cases. A subgroup of patients with possible/probable aspergillosis was defined by culture, microscopic, and positive galactomannan (GM) results as per the latest European Organization for Research and Treatment of Cancer/Invasive Infectious Diseases Study Mycoses Group (EORTC/MSG) criteria [30]. A patient is considered to have possible IA if a new and otherwise unexplained well-defined intrapulmonary nodule (with or without halo sign), an air crescent sign, or a cavity within an area of consolidation is radiologically documented. Probable IA is diagnosed when, on top of these radiological findings, microbiological proof of *A. fumigatus* infection is documented by galactomannan antigen detection (Platelia; Bio-Rad, Inc.) or positive cultures of *A. fumigatus*. Galactomannan was considered positive if at a level of ≥1.0 in BAL fluid or serum. For subjects with ABPA and CPA who received azole therapy, azole exposure was considered present when a subject was treated for more than 2 weeks with ITC or VRC. Prolonged exposure was considered if patients were on azoles within the last two years. Azoles including ITC and or VRC was the line of treatment for ABPA and CPA patients included in the present study. Resistance data obtained in this study were not used for clinical decision making.

About 10 mL of BAL specimens were sent to the laboratory for analysis. Samples were split for fungal culture, microscopy, and DNA extraction in a laminar airflow cabinet and an aliquot for PCR testing was frozen immediately. Clinical data were retrieved from the record systems. The diagnosis of CPA was based on antibody and radiology data [31], ABPA on clinical and serological data [32,33] and SAFS as described previously [34]. Another subgroup of patients with proven/probable aspergillosis was defined by culture, microscopic, and positive galactomannan (GM) results. a) DNA extraction from BAL fluid: DNA extraction was done in the laminar flow to avoid contamination. 200 µL of BAL samples were mixed with 250 µL of GF-1 tissues lysis buffer and 20 µL of proteinase K (GF-1 Tissues Extraction Kit, Vivantis Technologies Sdn Bhd, Malaysia) followed by pulsed-vortexing to obtain a homogenous solution. Briefly, 12 µL of GF-1 lysis enhancer were added to the homogeneous solution obtained in the previous step followed by gentle mixing and incubation at 65 °C for 13 h with occasionally mixing during incubation to ensure thorough digestion of the BAL sample. A further 20 µL of RNase A (20 mg/mL) were added, mixed and incubated at 37 °C for 10 min. Approximately two volumes of GF-1 tissue genomic DNA binding buffer were added and mixed thoroughly by pulsed-vortexing until a homogeneous solution is obtained followed by incubation for 10 min at 65 °C. We added 200 µL of absolute ethanol and mixed this immediately and thoroughly by pulsed vortexing to obtain a homogeneous solution. A further 650 µL of the sample were transferred to a GF-1 column assembled in a clean collection tube and centrifuged at 5000× *g* for 1 min and the flow through were discarded. The above step was repeated for remaining solution. The column was washed by adding 650 µL of GF-1 washing buffer followed by centrifugation at 5000× *g* for 1 min and the flow through were discarded. The washing of column was performed twice. Then, columns were dried by centrifuging at 10,000× *g* for 1 min to remove all the traces of ethanol. In addition, 200 µL of preheated GF-1 elution buffer were added to dried column, incubated at room temperature for 2 min followed by centrifugation at 5000× *g* for 1 min to elute the DNA. b) AsperGenius^®^ multiplex real-time PCR assay. The AsperGenius^®^ multiplex real-time PCR assay (PathoNostics, Maastricht, The Netherlands) was used to detect *Aspergillus* species and *Cyp51A* gene mutations. The species PCR allows for detection of *A. fumigatus* complex, *A. terreus* and *Aspergillus* species by targeting the 28S rRNA multicopy gene. The *A. fumigatus* probe detects the most relevant species of the *Fumigati* complex: *A. fumigatus*, *A. lentulus* and *A. felis*. The *Aspergillus* species probe specifically detects *A. fumigatus*, *A. terreus*, *A. flavus* and *A. niger*. An internal control is included to monitor for inhibition or manual handling errors. The resistance PCR targets the single-copy *Cyp51A* gene of *A. fumigatus* and detects TR_34_/L98H/Y121F/T289A mutations to differentiate the wild-type (WT) from mutant *A. fumigatus* via melting curve analysis. Additionally, a newly developed AsperGenius^®^ G54/M220 RUO multiplex real-time PCR assay, using melting-curve analyses was performed that detects G54 and M220 mutations in *Cyp51A* gene of *A. fumigatus.* The G54/M220 assays can detect all alterations in the G54 and M220 region and differentiate mutant from wild-type. Because the different alterations result in several similar melting curves the assay can only differentiate wild-type from mutant reliably but does not indicate substitution for example G54E or G54W. Each extracted BAL sample was tested in duplicate and a template control (blank) was included in each run to exclude contamination. A sample was considered positive when one of the duplicates showed increased fluorescence above the threshold. The positive control from the assay was used as a standard for the melting peaks and was tested simultaneously with the BAL samples to determine whether the melting peak represents WT or *A. fumigatus* with resistant associated mutations. The optimal *C*t cut-off and diagnostic performance was determined for the species probe of the PCR using the earlier *C*t value [4,12].

## 3. Results

**Figure 1:**
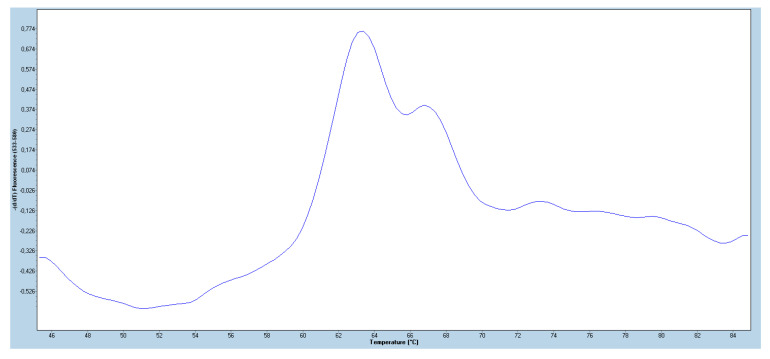
Melting curve analysis showing double peak due to mixed presence of wild-type and G54 mutant.

Of 160 BAL samples, the *A. fumigatus* species PCR was positive in 133 (83%). The optimal cycle threshold cut-off value for the AsperGenius^®^ Species real-time PCR was <36. With this cut-off, the PCR was positive in 133/160 (83%) BAL samples. Thirty-three patients (25%) had resistance-associated mutations and the remaining 100 patients harboured a WT genotype of *A. fumigatus*. The distribution of mutations in 33 patients included G54 with concomitant WT in 22 patients (67%), and TR_46_/Y121F/T289A with concomitant WT in 4 patients (12%). In the remaining seven patients, six harboured TR_34_/L98H (18%) mutations and one patient had M220 with concomitant WT genotype. Interestingly, except one all six patients with TR_34_/L98H genotype had mixed infections including TR_34_/L98H with wild-type genotype in three patients and in one each patient concomitant G54 + WT and TR_46_/Y121F/T289A + WT was recorded. Notably, 97% (32 out of 33) patients positive for resistance-associated mutations also had concomitant WT *A. fumigatus* suggesting mixed pulmonary infection/colonization (Table 1). A double peak in multiplex real-time PCR assay, clearly indicated a mixed presence of azole susceptible and azole-resistant *A. fumigatus* in BAL fluid samples (Figure 1).

Overall, only 23% (*n* = 37/160) of BAL samples yielded growth in culture as compared to 83% positive for *A. fumigatus* species PCR. A total of 37 *A. fumigatus* grew from equal numbers of patient BAL fluid specimens. The distribution of culture positivity is detailed in Table 1. Out of 33 patients who had resistance-associated mutations, only 27% (9/33) grew *A. fumigatus* in culture. A preliminary detection of azole resistance in all 37 *A. fumigatus* isolates recovered in culture by screening agar plates identified only two isolates that grew on ITC- (4 mg/L) and VRC- (1 mg/L) supplemented SDA plates. One grew on both ITC- and VRC-supplemented SDA plates while the other grew only on ITC-supplemented SDA plates.

The isolate that grew on ITC- and VRC-supplemented SDA plates showed a pan-azole resistant phenotype exhibiting elevated MICs to three triazoles namely, ITC (>16 mg/L), VRC (2 mg/L) and ISA (2 mg/L) and harboured the TR_34_/L98H mutation detected by *Cyp51A* gene sequencing as well as by AsperGenius^®^ Resistance multiplex real-time PCR assay. However, another isolate that grew only on ITC supplemented SDA plate exhibited high MIC to ITC (4 mg/L) and POS (1 mg/L) and harboured a G54E mutation which was confirmed by *Cyp51A* gene sequencing as well as by G54/M220 RUO real-time PCR assay. The range of MIC values of the remaining 35 culture positive *A. fumigatus* isolates was below the ECVs, recommended by CLSI M59-Ed2, of all the tested azoles and were as follow: ITC (0.25–0.5 mg/L), VRC (0.06–0.125 mg/L), ISA (0.06–0.25 mg/L) and POS (0.03–0.25 mg/L).

In this series, a total of 160 patients were included. The underlying conditions in 133 patients with *A. fumigatus* species positive PCR were as follows: CPA (total *n* = 27; WT = 11, resistance-associated mutations = 16), ABPA (*n* = 21; WT = 12, resistance-associated mutations = 9), severe asthma (*n* = 9; WT = 8, resistance-associated mutations =1), probable IA (*n* = 6; WT = 2, resistance-associated mutations = 4), possible IA (*n* = 5; WT = 2, resistance-associated mutations =3), lung tumour (*n* = 9; WT = 9), pulmonary emphysema (*n* = 9; WT = 9), previous thoracic surgery (*n* = 6; WT = 6), bronchiectasis (*n* = 6; WT = 6), interstitial pneumonia (*n* = 13; WT = 13), pulmonary tuberculosis (*n* = 15; WT = 15), non-tuberculous mycobacterium infection (*n* = 2; WT = 2), chronic obstructive pulmonary disease (COPD; *n* = 5; WT = 5). Patients whose BAL samples were negative for *A. fumigatus* (*n* = 27) had the following underlying diseases: COPD (*n* = 8), pulmonary tumor (*n* = 8), tuberculosis (*n* = 5), interstitial lung disease (*n* = 5) and one patient had a non-tuberculous mycobacterium infection. The clinical diagnosis of patients infected with *A. fumigatus* having resistance mutations included CPA (*n* = 16), ABPA (*n* = 9), probable IA (*n* = 4), possible IA (*n* = 3) and a single patient had SAFS. Azole exposure was present in 13 of 21 (62%) ABPA patients who were on ITC treatment for the previous 2–3 months and 10 out of 13 exposed had a prolonged history of previous courses of ITC within the last two years. Similarly, in CPA patients 78% (21/27) subjects had received previous treatment with azoles including 16 who were currently treated with VRC (in the previous 1–2 months) and had previously received ITC of 4–6 months duration. The remaining five had received only VRC for 2–4 months. Azole-resistant *A. fumigatus* isolates were found in 9/13 (69%) and 16/21 (76%) of ABPA and CPA patients exposed to ITC within the previous 3 years respectively. Notably in ITC exposed ABPA patients, 67% (6/9) showed the presence of the G54 mutation. The remaining three patients had environmentally derived mutations i.e., TR_34_/L98H in two patients and TR_46_/Y121F/T289A in a single patient. Similarly, in 87.5% (14/16) of ITC-exposed CPA patients G54 was the predominant mutation noted. Interestingly, of the remaining two CPA patients one exhibited the G54 mutation concomitant with TR_34_/L98H and one patient had a M220 mutation combined with a WT. G54 was also found in one patient each with SAFS and possible IA, both patients had a previous history of exposure to ITC in the previous three years. TR_46_/Y121F/T289A and TR_34_/L98H were common mutations observed in both probable (*n* = 4/4) and possible (*n* = 2/3) IA patients. Interestingly a single patient with possible IA had concomitant infection with both environmentally derived mutations i.e., TR_46_/Y121F/T289A and TR_34_/L98H. Among seven cases with possible/probable IA, a history of exposure to azoles was available in three patients. Two of the three patients had ITC exposure in the previous two months. The treatment data of patients with possible (*n* = 5) and probable IA (*n* = 6) were available for 8 patients (probable IA *n* = 5, possible IA *n* = 3). Of these 8 patients, three patients had resistance-associated mutations (probable IA *n* = 2; possible IA *n*= 1) and 5 patients had infection with WT genotype. Of three patients who had infection with resistance-associated mutations, one received conventional amphotericin B for a period of 10 days and expired. The remaining two received liposomal amphotericin B for two weeks followed by VRC for 4 weeks. Both patients were discharged and were not available for follow up. In the remaining 5 patients with the WT genotype, conventional and liposomal amphotericin B was given in one patient each (2 weeks) and the remaining three received VRC therapy for 2–4 weeks. Three of the five patients including two on amphotericin B therapy and one on VRC therapy died within 10–31 days of treatment.

## 4. Discussion

In this study, we demonstrate an alarmingly high frequency (25%) of azole resistance among patients with chronic and allergic bronchopulmonary aspergillosis. AsperGenius^®^ Resistance multiplex and AsperGenius^®^ G54/M220 RUO real time PCR assays detected four mutations (TR_34_/L98H, TR_46_/Y121F/T289A, G54 and M220) that confer azole resistance in *A. fumigatus* directly in 59% of BAL samples of patients with CPA and in 43% of patients with ABPA. Furthermore, in 97% of patients this molecular assay simultaneously detected mixed population of both susceptible and resistant *A. fumigatus* in the same BAL samples. AsperGenius^®^ Resistance multiplex real-time PCR assay that directly detects environmentally driven mutations i.e., TR_34_/L98H and TR_46_/Y121F/T289A is fully validated for in vitro diagnostic testing of BAL fluid samples [4,9,10,13,14,15]. As yet only three commercial kits are currently available (AsperGenius^®^, PathoNostics, Maastricht, Netherlands, MycoGenie^®^, Ademtech, Pessac, France and Fungiplex^®^ Aspergillus Azole-R IVD PCR, Bruker Daltonik GmbH, Bremen, Germany), which are designed to identify the promoter region insertion polymorphisms TR_34_/L98H and TR_46_/Y121F/T289A and specific alleles [4,12,13,14,15,16,17,18,19,20,21,22]. Direct detection assays targeting polymorphisms in the *Cyp51A* open reading frame (ORF, e.g., G54, M220, G138, G448) have been applied in limited studies [35,36,37]. The present study applied for the first time a new AsperGenius^®^ M220/G54 RUO real time PCR assay in detecting azole resistance in high risk patients with chronic *Aspergillus* diseases while on azole therapy. The specialized Chest Hospital, Delhi, India, caters to chronic respiratory disease patients mostly other than tuberculosis. Specifically, mutations at position G54 are known to be the consequence of long-term azole therapy in patients with chronic aspergillosis. [38,39]. AsperGenius^®^ G54/M220 RUO real-time PCR assay identified G54 as the single most significant mutation present in 67% of all 33 patients harbouring resistance-associated mutations in BAL samples. The high percentage of patients harbouring resistance-associated mutations is probably linked to itraconazole exposure which was considerably high in the patient population investigated in the present study being present in 62% of ABPA and 78% of CPA patients. Interestingly, the G54 mutation was the sole resistance mechanism in 87.5% of 16 patients with CPA. Similarly, G54 was also the predominant mutation in 67% of nine patients with ABPA. Azole resistance has been reported to emerge in patients with chronic pulmonary aspergillosis and pulmonary aspergilloma during azole therapy [40]. It was previously suggested that in the case of aspergilloma or cavitary aspergillus disease, the fungus is able to undergo multiple generations in the patient by asexual reproduction. Sporulation (in the lung) as opposed to hyphal growth may be important to facilitate the expression of the azole-resistant phenotype [41].

High rates of azole-resistant *A. fumigatus* isolates in adult cystic fibrosis (CF) subjects has been shown to be associated with previous ITC exposure. Azole-resistant *A. fumigatus* isolates were found in 5/25 (20%) adult CF subjects treated with ITC within the previous 3 years [42]. Similar finding has been reported by Mortensen et al., who found that 6 patients, previously exposed to ITC, were infected with *A. fumigatus* that had reduced susceptibility to ITC [43]. The limitation of the present study is that in view of limited resources in a general public hospital in Delhi, India, we were unable to perform therapeutic drug monitoring of patients on ITC therapy. Because azole plasma concentrations may be widely variable, the role of pharmacokinetic variability in the reduced azole susceptibility of *A. fumigatus* isolates in the present study could not be ascertained. Low plasma concentrations of ITC have been noted in subjects with azole-resistant *A. fumigatus* in previous studies [25,42] but the number of patients analysed in these studies were too small for us to draw significant conclusions regarding the effect of long-term underdosing on resistance induction in *A. fumigatus*. It is pertinent to mention here that the quality of ITC formulations related to the proprietary formulation of ITC can also have a significant influence on its efficacy, thus affecting the final therapeutic outcome and in some cases leading to treatment failure [44,45]. A recent study from India showed a marked variation in the quality of ITC pellets that determine the absorption profile and thereby the reduced therapeutic efficacy of several ITC formulations [46]. In the present study, we were unable to monitor the quality of ITC formulations as antifungals were procured by patients. The effect of ITC formulation to achieve optimal or suboptimal efficacy and subsequent development of resistance warrants further studies. However, another factor to be taken into consideration is the presence of G54 mutations in the local agricultural environment [47]. Interestingly, we have previously reported G54E mutation in environmental soil samples in India, Romania and Tanzania [8]. Two recent publications from Germany and Switzerland also reported the presence of G54 single substitution mutation (G54R) in environmental *A. fumigatus* isolates [9,10]. The possibility that patients may acquire this mutation from environmental sources is probably another important route leading to colonisation/infection in our patient population.

Furthermore, both TR_34_/L98H and TR_46_/Y121F/T289A are the predominant resistance mechanisms in the environment of India. We have previously reported TR_34_/L98H resistance mechanism in 7% of environmental samples in India. Interestingly, all Indian environmental and clinical azole-resistant *A. fumigatus* isolates carrying TR_34_/L98H shared the same microsatellite genotype suggesting environmental route of transmission in Indian patients [27]. Also, previously we have documented in an environmental survey that both TR_34_/L98H and TR_46_/Y121F/T289A resistance mechanisms coexist in Indian agricultural fields [48]. In the present study co-infection with both TR_34_/L98H and TR_46_/Y121F/T289A environmental mutations was noted in a single case with possible IA. Also, a CPA patient had concomitant presence of both TR_34_/L98H and G54 in the lung. Both these cases strongly indicated that multiple resistant genotypes of *A. fumigatus* strains with several different resistance mechanisms may be inhaled by patients and subsequently selected in the lung. Another matter of concern in this study was the presence of both WT and azole-resistant *A. fumigatus* in same BAL specimens in 97% of subjects. The majority of patients had chronic diseases such as tuberculosis, bronchiectasis, and COPD suggesting *A. fumigatus* colonization/inhalation in the compromised lungs. Importantly, in patients with confirmed diagnosis of probable and possible IA both WT and resistant genotypes were noted. Previously, in three patients with IA simultaneous presence of WT *A. fumigatus* and *A. fumigatus* with mutant genes (two patients with TR_34_/L98H mutation and one patient with TR_46_/T289A/Y121F mutation) and therefore mixed infections with azole-susceptible and resistant isolates has been confirmed by the AsperGenius^®^ assay [15]. *Aspergillus fumigatus* is genetically diverse and multiple genotypically different isolates can be obtained from respiratory samples from single patients [49].

A low culture yield of 23% compared to 83% positivity with *A. fumigatus* species PCR highlights concerns of low yield of routine microbiology cultures. In culture- negative, PCR-positive samples, we detected azole-resistant mutations in azole target *Cyp51A* gene in 34% (33/96) of BAL samples. In fact, *A. fumigatus* grew in only 9 of these 33 samples that were positive for resistance-associated mutations by PCR. Furthermore, azole resistance may occur due to several non-*Cyp51A* gene mediated resistance mechanism and several other species of *Aspergillus* may show high azole resistance. Both of these scenarios were not detected in the present study. Unfortunately, by using a screening method with azole supplemented plates we could identify only two *A. fumigatus* isolates that had confirmed resistance-associated mutations by *Cyp51A* sequencing. Culture of respiratory samples is a slow and insensitive method for the diagnosis of aspergillosis, although its sensitivity can be improved by increasing the culture volume. In a recent study positivity rate of conventional cultures of respiratory specimens yielded fungal growth in 15.7% and that of high-volume culture (HVC) was 54.2% [50]. Furthermore, the authors reported that HVC allows for detection of azole-resistant isolates that would have been missed by conventional culture. Our low culture-positivity of azole-resistant isolates could be attributed to the conventional culture methods used. Furthermore, a mixed population of azole-susceptible and azole-resistant isolates was present in 97% of BAL specimens as detected by PCR. We believe that screening single colony of *A. fumigatus* for azole resistance on azole supplemented agar plates resulted in detection low rates of azole resistance in culture. Therefore, a significant number of cases involving azole-resistant isolates may go unnoticed. It is important to emphasize that in geographical regions where azole resistance is common, it is recommended to test multiple and preferably all separate *A. fumigatus* colonies for azole resistance. A limitation of this study is that therapeutic outcome data were not available. As the majority of patients with resistance-associated mutations had CPA and ABPA, both clinical conditions required multiple hospitalizations often in different hospitals, consequently on several occasions’ patients could not be followed up. Finally, these data suggest a major role of a single substitution mutation in high azole resistance in CPA and ABPA patients in India. Detection of the true burden of azole resistance is greatly hampered by conventional culture techniques and warrant standardization of respiratory culture and inclusion of rapid techniques to detect resistance markers directly from respiratory samples.

## Figures and Tables

**Table 1:** Details of patients (*n* = 33) positive for resistance-associated mutations detected in bronchoalveolar lavage (BAL) specimens by AsperGenius^®^ Resistance multiplex real-time polymerase chain reaction (PCR) assay and a newly developed AsperGenius^®^ G54/M220 RUO multiplex real-time PCR assay.

Resistance-Associated Mutations	Number of Patients	Diagnosis (no. of Patients)	No. of Culture Positive BAL Samples for *A. fumigatus* (no. of Patients)
G54 ^#^ + WT	22	CPA (*n* = 14); ABPA (*n* = 6); SAFS (*n* = 1); Possible IA (*n* = 1)	5 * (CPA = 3, ABPA = 1, possible IA = 1)
TR_46_/Y121F/T289A + WT	4	Probable IA (*n* = 2); Possible IA (*n* = 1); ABPA (*n* = 1)	1 WT (possible IA)
TR_34_/L98H + WT	3	ABPA (*n* = 1); Probable IA (*n* = 2)	1 ** (probable IA)
TR_34_/L98H	1	ABPA (*n* = 1)	1WT
TR_34_/L98H + G54 + WT	1	CPA (*n* = 1)	Negative
M220 ^#^ + WT	1	CPA (*n* = 1)	Negative
TR_34_/L98H + TR_46_/Y121F/T289A + WT	1	Possible IA (*n* = 1)	1WT
WT	100		28

CPA, chronic pulmonary aspergillosis; ABPA, allergic bronchopulmonary aspergillosis; SAFS, severe asthma with fungal sensitization; WT, wild-type *Aspergillus fumigatus.* # G54 and M220 detected by AsperGenius^®^ G54/M220 RUO multiplex real-time PCR assay in BAL specimens. * G54 mutation identified by *Cyp51A* gene sequencing of culture isolate in one patient with CPA. ** TR_34_/L98H mutation identified by *Cyp51A* sequencing of culture isolate in one patient with probable IA.
